# Optimizing the performance of Au_*y*_/Ni_*x*_/TiO_2_NTs photoanodes for photoelectrochemical water splitting†

**DOI:** 10.1039/d3ra02011h

**Published:** 2023-05-09

**Authors:** Shaimaa K. Mohamed, Amany M. A. Bashat, Hassan M. A. Hassan, Nahla Ismail, Waleed M. A. El Rouby

**Affiliations:** a Department of Chemistry, Faculty of Science, Suez University 43518 Suez Egypt Shaimaa.Mohamed@Sci.suezuni.edu.eg h.hassan@suezuniv.edu.eg; b Physical Chemistry Department, Centre of Excellence for Advanced Sciences, Renewable Energy Group, National Research Centre Dokki 12311 Giza Egypt; c Materials Science and Nanotechnology Department, Faculty of Postgraduate Studies for Advanced Science (PSAS), Beni-Suef University 62511 Beni-Suef Egypt waleedmohamedali@psas.bsu.edu.eg

## Abstract

Water splitting using photoelectrochemical (PEC) techniques is thought to be a potential method for creating green hydrogen as a sustainable energy source. How to create extremely effective electrode materials is a pressing concern in this area. In this work, a series of Ni_*x*_/TiO_2_ anodized nanotubes (NTs) and Au_*y*_/Ni_*x*_/TiO_2_NTs photoanodes were prepared by electrodeposition *via* cyclic voltammetry and UV-photoreduction, respectively. The photoanodes were characterized by several structural, morphological, and optical techniques and their performance in PEC water-splitting for oxygen evolution reaction (OER) under simulated solar light was investigated. The obtained results revealed the nanotubular structure of TiO_2_NTs was preserved after deposition of NiO and Au nanoparticles while the band gap energy was reduced allowing for effective utilization of solar light with lower charge recombination rate. The PEC performance was monitored and it was found that the photocurrent densities of Ni_20_/TiO_2_NTs and Au_30_/Ni_20_/TiO_2_NTs were 1.75-fold and 3.25-fold that of pristine TiO_2_NTs, respectively. It was confirmed that the performance of the photoanodes depends on the number of electrodeposition cycles and duration of photoreduction of gold salt solution. The observed enhanced OER activity of Au_30_/Ni_20_/TiO_2_NTs could be attributed to the synergism between the local surface plasmon resonance (LSPR) effect of nanometric gold which increased solar light harvesting and the p–n heterojunction formed at the NiO/TiO_2_ interface which led to better charge separation and transportation suggesting its potential application as an efficient and stable photoanode in PEC water splitting for H_2_ production.

## Abstract 2

Water splitting using photoelectrochemical (PEC) techniques is thought to be a potential method for creating green hydrogen as a sustainable energy source.

## Graphical abstract

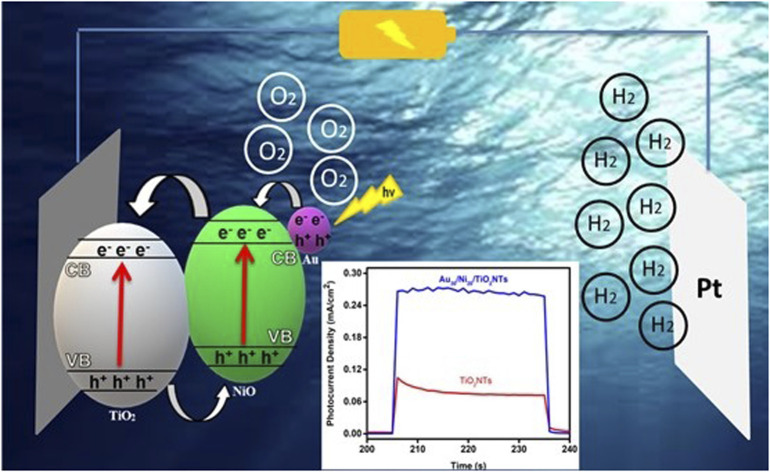

## 1. Introduction

Developing renewable, sustainable, and clean energy resources continues to be at the core of scientific research nowadays to address the growing problems of energy demand and environmental pollution.^1–4^ One of the most promising green strategies is utilization of solar energy in the production of hydrogen gas by PEC water splitting as it can store the renewable and clean solar energy in chemical bonds while operating at low input bias and room temperature.^1,4,5^ In addition, hydrogen is considered an ideal energy carrier due to its renewability, cleanliness, high energy density and zero CO_2_ emissions.^6–9^ The efficiency of PEC water splitting is greatly dependent on the following crucial factors: (i) efficient utilization of solar energy *via* minimizing the band gap of the semiconductor photoelectrode; (ii) the charge transfer rate to the photoelectrode/electrolyte interface and (iii) rapid surface reaction between the photogenerated electrons/holes and the electrolyte.^10^ Current research is focusing on enhancing the efficiency of the process through controlling the photoelectrode morphology, tuning the band gap, optimizing electrolyte conditions, and engineering of designed interfaces.^11,12^

One of the most employed photoanodes is TiO_2_ nanotube arrays (TiO_2_NTs) which stands out other titania structures due to stability, highly oriented electron transportation channels and its larger surface area resulting in enhanced diffusion of light, photogenerated carriers and reactants through the entire tubular depth.^13,14^ However, large bandgap implying weak solar light absorption and high carrier recombination rates are major drawbacks which can be overcome by chemical modification in the design of the photoanode material.^15^ The modification strategies may be directed to band gap reduction *via* chemical doping,^16^ retardation of charge recombination by heterojunction formation with other materials^17,18^ and enhancing solar energy harvesting through surface sensitization.^19–21^

It is widely acknowledged that doping of transition metal ions can help reducing the band gap thereby extended solar energy absorption to the visible range while acting as a trap for the photogenerated electrons thus suppressing the carrier recombination rate.^22–24^ Ni or Ni – related species (*i.e.* NiO, NiOOH, Ni–NiO) were reported to boost the photocatalytic activity of titania towards H_2_ production with a relative uncertainty about the active reaction center (Ni^0^ or Ni^2+^).^25–32^ Dong *et al.*^30^ prepared Ni-doped TiO_2_ nanotubes by anodizing Ti–Ni alloys and found that the photoconversion efficiency of PEC water splitting was enhanced 3.35 times the undoped titania which was attributed to improved light absorption and facilitated separation of photogenerated electron–hole pair. Li *et al.*^33^ contributed the enhanced photocatalytic and photoelectrochemical activity of NiO/TiO_2_ hollow microspheres to the construction of p–n junctions with an inner electric field between TiO_2_ and NiO.

Localized surface plasmonic resonance effect of nanometric gold has been reported to enhance PEC water splitting *via* facilitating oxygen evolution reaction.^14,34^ Through LSPR the interactions between irradiation photons and Au atoms are enhanced thus increasing visible light absorption on semiconductor surface and reducing diffusion distance of the photogenerated holes to the electrode/electrolyte interface.^34^

Improvement of PEC water-splitting performance can be expected by co-doping transition metal and plasmonic material like Au due to synergistic effects.^34^ Oros-Ruiz *et al.*^35^ found that Au–NiO/TiO_2_–P25 photocatalyst prepared by deposition–precipitation method were superior to pristine titania in the photocatalytic water splitting reaction for hydrogen production. However, doping of TiO_2_NTs with both NiO and Au and utilization of the prepared nanotubular photoanode in PEC water-splitting for H_2_ production is seldom reported. In this work, highly ordered TiO_2_NTs were synthesized by anodization method and a series of Ni_*x*_/TiO_2_NTs photoanodes were prepared through electrodeposition. Another series of Au_*y*_/Ni_*x*_/TiO_2_NTs photoanodes were prepared by facile and simple photoreduction method. The performance of the prepared photoelectrodes was investigated in PEC water-splitting for H_2_ production under simulated sun light.

## 2. Materials and methods

### 2.1 Materials

A titanium plate (99.9%) was purchased from AMERICAN ELEMENTS (USA). Ammonium fluoride (95%) and nickel chloride (98%) were purchased from Winlab laboratory chemicals reagents fine chemicals (United Kingdom). Gold(iii) chloride trihydrate (HAuCl_4_·3H_2_O), acetone (≥99.8) and ethanol (≥99.5%) were purchased from (Sigma Aldrich, Germany). Tri-sodium citrate (C_6_H_5_O_7_Na_3_·2H_2_O) was purchased from (Qualikems, India). Ethylene glycol (99.9%) was obtained from (Pharmco Product Inc., North America). Sodium sulphate (Oxford Laboratory Reagents, India). All chemicals were utilized without further purification. All the solutions were prepared by deionized water.

### 2.2 Synthesis of the photoelectrodes

#### 2.2.1 Fabrication of TiO_2_NTs photoanode

TiO_2_NTs were fabricated through anodization of titanium foil (2 × 1.5 cm). The titanium foils were ultrasonically cleaned with acetone, ethanol and deionized water for 30 min sequentially and dried in N_2_ stream for 3 min. The anodization of the cleaned titanium foil was performed in a mixture of ethylene glycol, 0.3 wt% ammonium fluoride and 2 wt% deionized water at 40 V for 1 h. Then, the anodized Ti foil was ultra-sonicated with ethanol and annealed in air at temperature of 450 °C for 3 h with a heating rate of 5 °C min^−1^.

#### 2.2.2 Fabrication of Ni_*x*_/TiO_2_NTs photoanodes

The prepared TiO_2_NTs was dipped in 50 mL of 0.05 M NiCl_2_ electrolyte solution in a conventional electrochemical cell with a three-electrode system, containing a platinum electrode as the counter electrode and an Ag/AgCl electrode as the reference electrode. The cyclic voltammetry method was utilized in the negative potential range of −0.1 to −0.5 V at scanning rate 10 mV s^−1^ to obtain the Ni_*x*_/TiO_2_ NTs photoanodes at different number of cycles (where *x* represents the number of cycles = 3, 5, 10, 20 or 30). The prepared photoelectrodes were dried at 80 °C for 2 h and annealed in air at temperature of 450 °C for 3 h with a heating rate of 5 °C min^−1^ to obtain the NiO phase.

#### 2.2.3 Fabrication of (Au_*y*_/Ni_20_/TiO_2_NTs) photoanodes

**Fig. 1:**
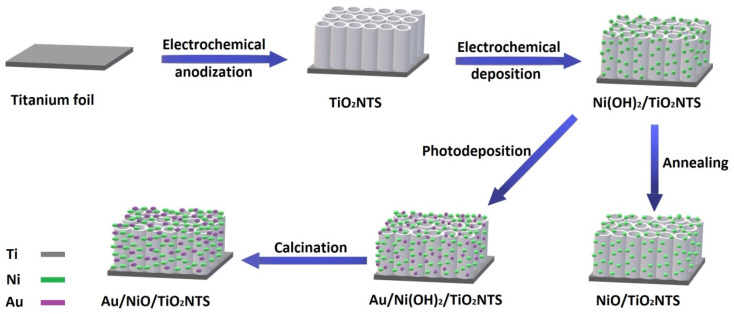
Scheme of the procedure for the preparation of Ni_*x*_/TiO_2_NTs and Au_*y*_/Ni_*x*_/TiO_2_NTs photoanodes.

The photoelectrode Ni_20_/TiO_2_NTs was selected to continue the fabrication process based on its performance in the PEC water-splitting reaction. A series of Au_*y*_/Ni_20_/TiO_2_ NTs photoelectrodes were prepared by photoreduction under UV irradiation (where *y* represents irradiation duration = 15, 30, 60 or 120 min). Un-annealed Ni_20_/TiO_2_ NTs was immersed in solution of 0.05 mM HAuCl_4_ and 1 mM tri-sodium citrate which act as capping and reducing agent and was irradiated with UV light (*λ* = 365 nm) for different durations. After washing with deionized water, the product was calcined in air at temperature of 450 °C for 2 h with a heating rate of 2 °C min^−1^. The fabrication process scheme is summarized in Fig. 1.

### 2.3 Characterization of the prepared materials

A field emission scanning electron microscope (FE-SEM, ZEISS Sigma 500VP) was used for checking the surface morphologies of the different samples. Elemental analysis was performed by energy-dispersive X-ray spectroscopy (EDS) analysis using equipment fixed on the SEM and operated with an acceleration voltage of 15 kV. High-resolution transmission electron microscope (HRTEM) images were taken by JEOL JEM-2100 electron microscope, where sample was prepared for analysis by scratching from titanium substrate, then ultrasonicated with ethanol. X-ray diffraction (XRD) was performed using a (Bruker D8 Advance, Germany) X-ray powder diffractometer, using Cu Kα radiation (wavelength 1.54 A, 40 mA, 45 kV). The Shimadzu 2600 spectrophotometer, Japan with Ba_2_SO_4_ as the reflectance standard was used for UV-vis analysis. Photoluminescence (PL) spectra were measured using JASCO (FP-8300) spectrofluorometer.

### 2.4 Photoelectrochemical measurements

The photoelectrochemical measurements were performed by Biologic SP-150 potentiostat. The performance of the fabricated photoelectrodes in PEC water-splitting was investigated by its utilization as the working electrode (1.5 cm × 1 cm) in a three-electrode configuration cell with Pt electrode as a counter electrode and Ag/AgCl reference electrode where 0.5 M Na_2_SO_4_ solution (pH ∼ 7) served as the electrolyte. The working photoelectrode was illuminated by xenon lamp-simulated solar light (100 mW, 1.5 AM). Three techniques were used to monitor the performance namely, liner sweep voltammogram (LSV), chronoamperometric measurements, and electrochemical impedance spectroscopy (EIS). Linear sweep voltammetry was measured in the voltage window starting from −0.6 V to +1 V at a scan rate of 10 mV s^−1^ and under chopped applied (ON/OFF) cycles with intervals of 5 s in the dark and 5 s under illumination. Chronoamperometry was performed under chopped applied ON/OFF cycles with intervals of 30 s in the dark and 30 s under illumination and the measurements were performed at 1 V *vs.* Ag/AgCl under continuous simulated sunlight illumination for 6 minutes. The electrochemical impedance spectroscopy measurements were measured for frequency range 0.05 Hz to 100 KHz at potential of 0 V *vs.* Ag/AgCl electrode with an amplitude of 10 mV. The stability of the fabricated photoelectrodes was measured by chronoamperometry at 1 V (*vs.* Ag/AgCl) for 1 h in 0.5 M Na_2_SO_4_ solution.

## 3. Results and discussion

### 3.1 Structural, chemical, optical and morphological characterization of Au_*y*_/Ni_*x*_/TiO_2_NTs photoelectrodes

**Fig. 2:**
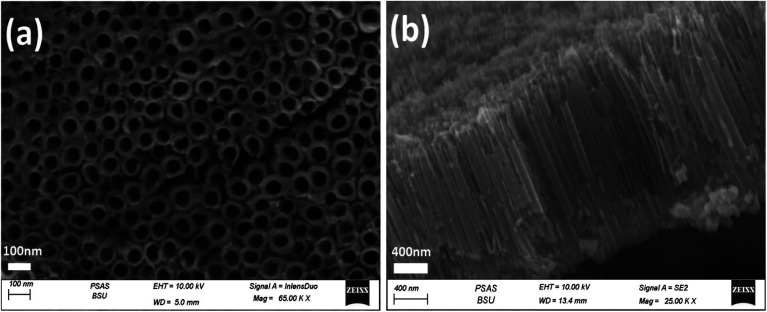
Top-view (a) and side-view (b) FESEM images of TiO_2_NTs photoanode.

**Fig. 3:**
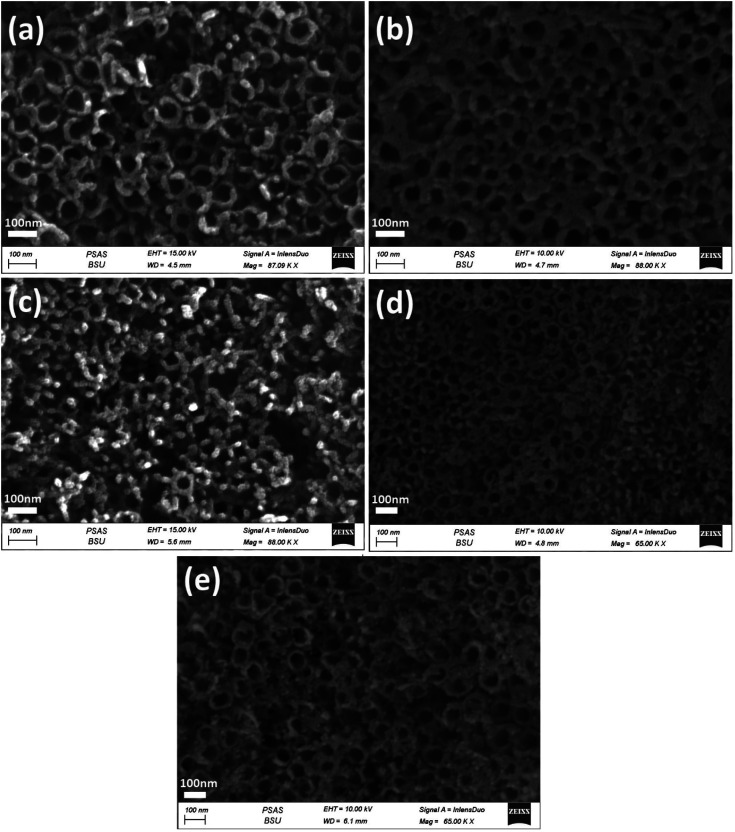
FESEM images of the top-view of Ni_*x*_/TiO_2_NTs photoanodes synthesised by different electrodeposition cycles (a) 3 cycles, (b) 5 cycles, (c) 10 cycles, (d) 20 cycles and (e) 30 cycles.

**Fig. 4:**
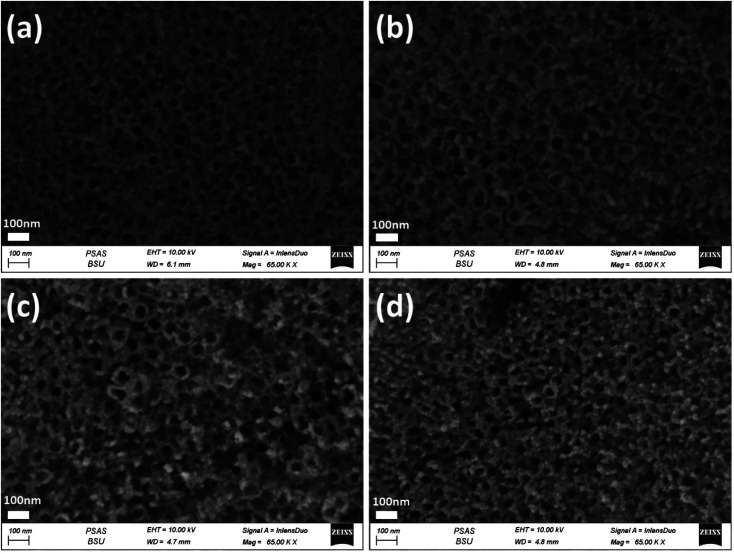
FESEM images of the top-view of Au_*y*_/Ni_20_/TiO_2_NTs photoanodes synthesised by different time intervals of photodeposition (a) 15 min, (b) 30 min, (c) 60 min and (d) 120 min.

The morphologies of the photoelectrodes are investigated by FESEM and are given in (Fig. 2–4). Fig. 2a and b present the top-view and cross-sectional FESEM images of TiO_2_NTs photoelectrode, respectively, which demonstrate the nanotube array uniformity with average diameter around 70 nm, the wall thickness 20 nm and the tubes length ranging from 1.4–2.0 μm. The length of the prepared TiO_2_NTs is favourable for the photoelectrocatalytic water splitting reaction because the maximum penetration depth of light in TiO_2_ nanotubes is 2.0 μm, tubes longer than 2.0 is expected to show higher electronic resistance which would retard the photogenerated carriers transportation rate^36,37^ and facilitate recombination of the photogenerated electron–hole pairs.^14^

Fig. 3a–e shows the Ni_*x*_/TiO_2_NTs photoelectrodes with NiO nanoparticles deposited onto the top and inner walls of TiO_2_NTs while the tubular morphology is preserved. The spacing among nanotubes is no longer obvious, and the nanotube inner diameter and wall become smaller and thicker due to the deposition of Ni species on the outer and inner surfaces of the tubes. The Ni_*x*_/TiO_2_NTs photoelectrode prepared at 30 cycles showed clogged tubes due to increasing amount of deposited NiO nanoparticles. The morphology of Au_*y*_/Ni_20_/TiO_2_NTs photoelectrodes was given by Fig. 4a–d. The images revealed that the photoreduction deposition of gold didn't change the morphology of tubes and a further clogging is observed with increasing the time intervals of reduction.

**Fig. 5:**
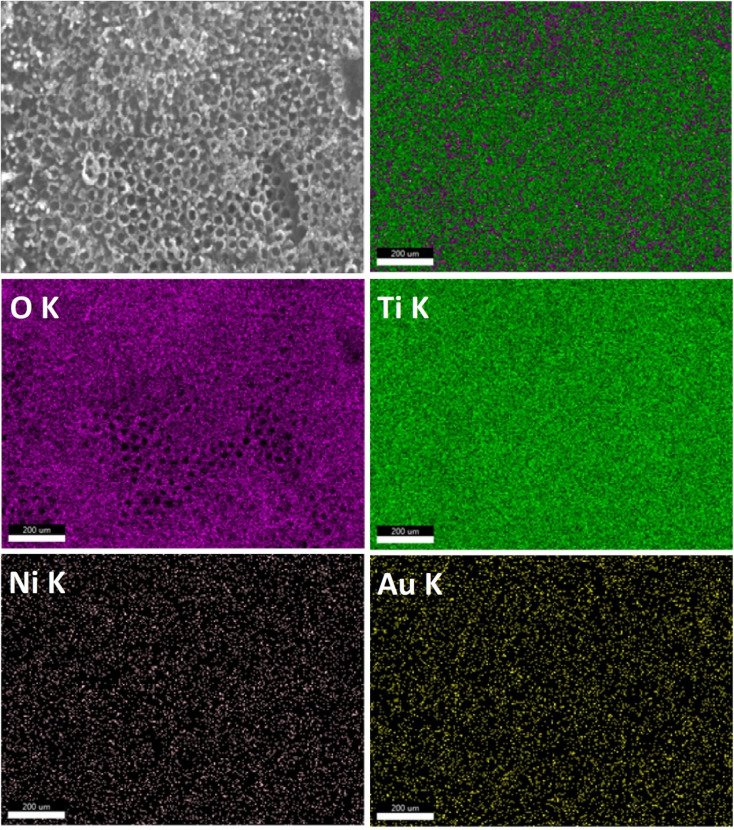
The EDS elemental mapping of Au_30_/Ni_20_/TiO_2_NTs.

The energy dispersive X-rays' analysis was performed for all fabricated photoelectrodes (Fig. S1–S10†), and the calculated element's atomic percent were presented in Table 1. The presence of the elements Ti, Ni and Au is confirmed in a manner corresponding to the preparation method. The amount of Ni was raised gradually by increasing the number of cycles of electrodeposition from 5 to 20 cycle. It was found in photoelectrodes containing gold that the amount of Au increased as the time intervals of photoreduction deposition increased. The EDS elemental mapping results is present in Fig. 5 for one selected photoelectrode (Au_30_/Ni_20_/TiO_2_NTs) where nickel (Ni), gold (Au) elements were uniformly and homogenously deposited on the surface of titania tubes. This would lead to well – dispersed active sites along the tubes which enhance the efficiency of the photoelectrocatalytic reaction.^38^

**Table 1:** Elemental composition and band gap values of the prepared photoanodes

Photoelectrode	Ti	O	Ni	Au	Band gap (eV)
TiO_2_	14.70	85.30			3.05
Ni_3_/TiO_2_NTs	26.19	73.47	0.34		2.85
Ni_5_/TiO_2_NTs	26.15	73.47	0.38		2.83
Ni_10_/TiO_2_NTs	26.98	72.60	0.42		2.82
Ni_20_/TiO_2_NTs	29.84	69.70	0.46		2.80
Ni_30_/TiO_2_NTs	12.69	86.86	0.45		2.81
Au_15_/Ni_20_/TiO_2_NTs	12.62	86.82	0.36	0.20	2.93
Au_30_/Ni_20_/TiO_2_NTs	14.39	84.96	0.42	0.23	2.89
Au_60_/Ni_20_/TiO_2_NTs	13.59	85.70	0.39	0.33	2.95
Au_120_/Ni_20_/TiO_2_NTs	13.10	86.03	0.51	0.37	2.96

**Fig. 6:**
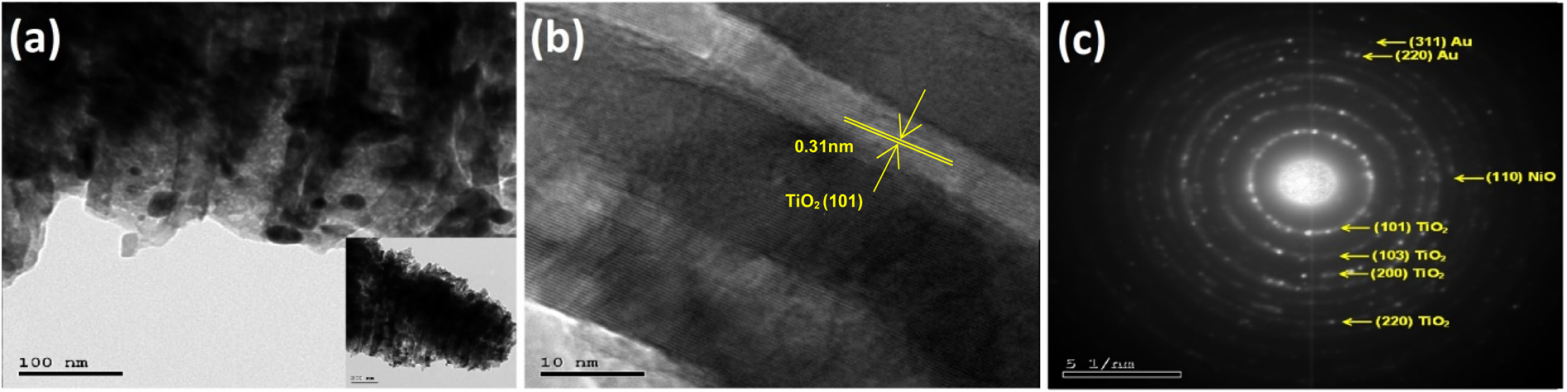
(a and b) HRTEM images of Au_30_/Ni_20_/TiO_2_NTs and (c) SAED patterns of photoanode.

HRTEM images for one selected photoelectrode Au_30_/Ni_20_/TiO_2_NTs showed in Fig. 6 confirmed the nano-tubular structure. The surface of TiO_2_NTs was rough where gold (Au) and nickel (NiO) particles can be found dispersed all over the surface of nanotubes. The lattice fringes of 0.31 nm corresponds to the reflection from the (101) plane of anatase TiO_2_ is identified as seen in Fig. 6b.^37^Fig. 6 c represented the selected area electron diffraction (SAED) which confirmed the crystalline structure of the photoelectrode and the presence of diffraction rings corresponding to (101), (103), (200), and (220) planes of TiO_2_.^39^ The (220) and (311) planes corresponded to metallic gold^40^ where the plane (110) may be indexed to NiO phase.^41^

**Fig. 7:**
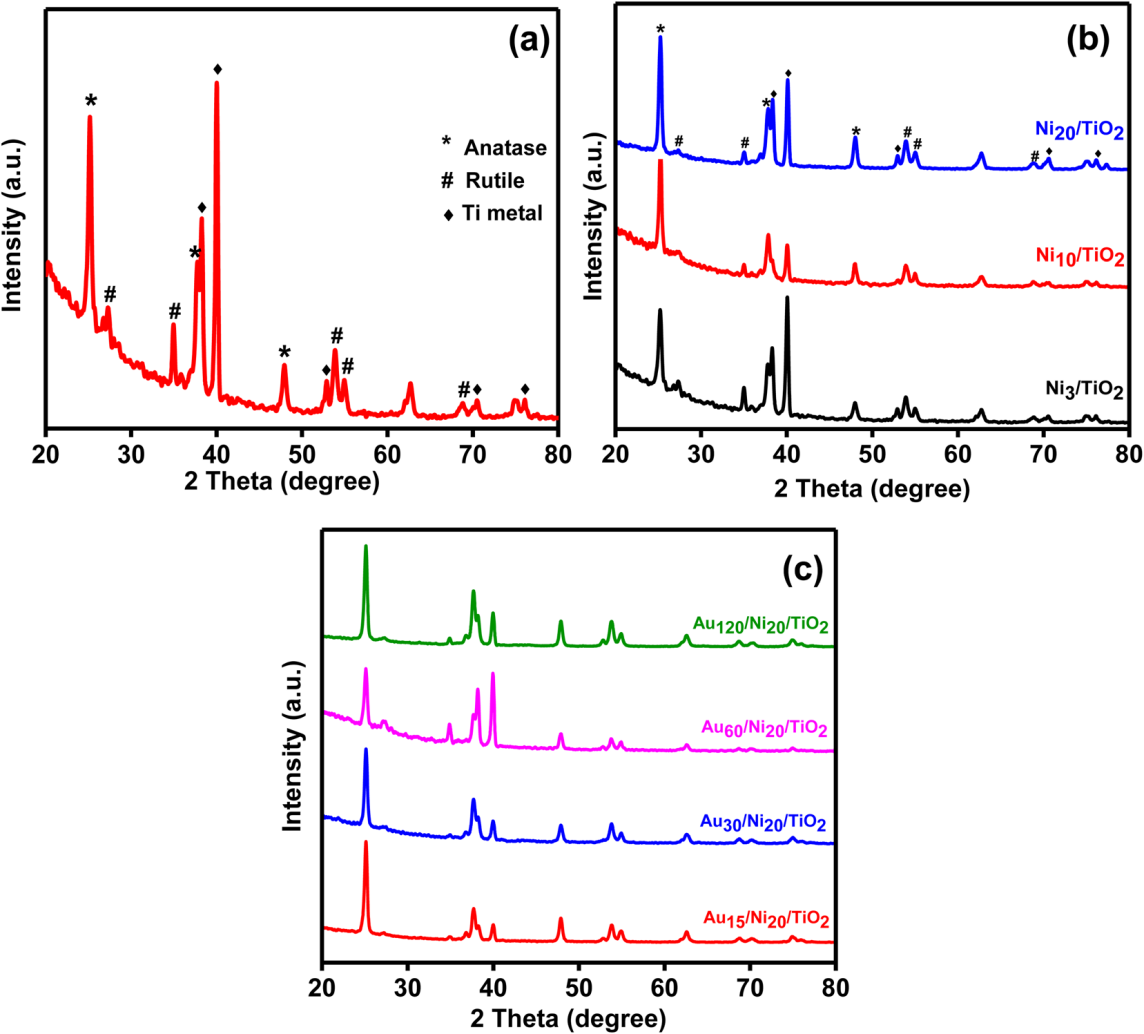
XRD diffractograms for the prepared TiO_2_NTs photoanode (a), Ni_*x*_/TiO_2_NTs photoanodes (b) and Au_*y*_/Ni_20_/TiO_2_NTs photoanodes (c).

The XRD diffractograms of the prepared photoelectrodes were given in Fig. 7. All prepared samples showed the presence of three phases which are metallic titanium, anatase and rutile. The peaks corresponding to anatase appeared at 2*θ* = 25.1°, 37.7° and 48° characteristic of (101), (004) and (200) planes, respectively (JCPDS 21-1272).^19,42^ The peaks at 2*θ* = 38.2°, 40°, 52.8°, 70.4° and 76.1° corresponded to (002), (101), (102), (103) and (112) planes, respectively of metallic titanium, (JCPDS 44-1294), attributed to the metallic substrate.^43^ Small peaks due to rutile as a minor phase was located at 2*θ* = 27.2°, 35°, 53.85°, 55° and 68.8° characteristic of (110), (101), (200), (211), and (220) planes (JCDPDP 01-089-8304).^44^ The anatase: rutile ratio was 2 : 1 in TiO_2_NTs sample suggesting enhanced performance in PEC due to improved hole transfer rate across TiO_2_NTs/electrolyte interface.^42,45^ The crystalline size of the anatase phase was found to be 25 nm confirming the nanostructure of the prepared tubes. There were no peaks detected for gold or nickel phases may be due to its low concentration^42,46^ and high dispersion. Another possible explanation may be provided by considering the limitations of X-ray powder diffraction technique.^47^ Amorphous phases do not give rise to diffraction peaks. Phase overlap may hinder phase identification while due to matrix effects, strongly diffracting phases would obscure weakly diffracting one. Thus, any of the above-mentioned reasons may account for the absence of diffraction peaks for gold and nickel – containing phases.

**Fig. 8:**
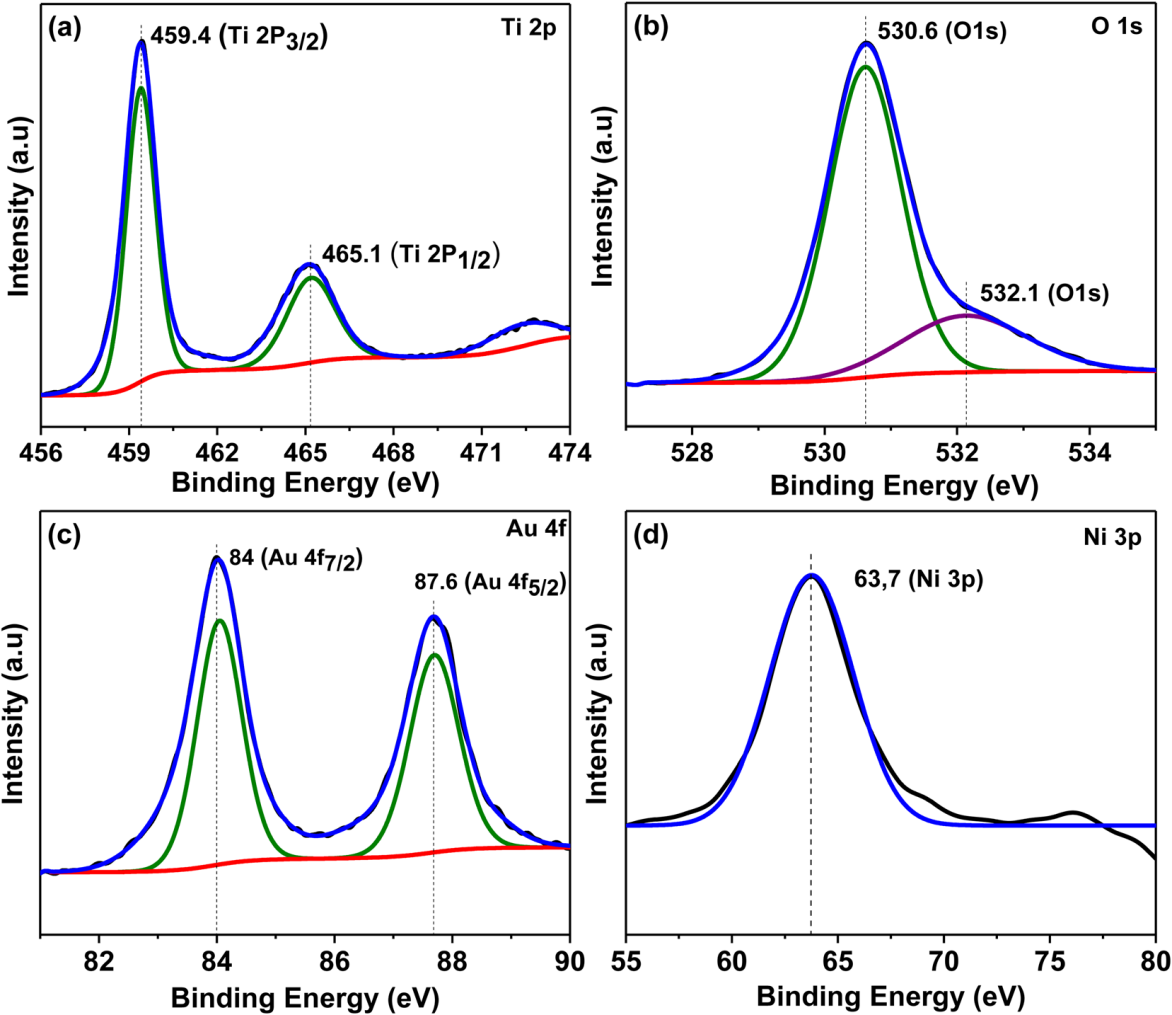
XPS spectra for Au_30_/Ni_20_/TiO_2_NTs photoelectrode (a) Ti 2p, (b) O 1s, (c) Au 4f and (d) Ni 3p.

Fig. 8 represents XPS spectra for Au_30_/Ni_20_/TiO_2_NTs photoelectrode which was analysed to determine surface chemical composition and oxidation states. Peaks at 459.4 and 465.1 eV of the titanium spectra corresponded to photoelectronic splitting of 2p_1/2_ and 2p_3/2_ Ti^4+^ orbitals.^48^ The XPS spectra for Au 4f showed two peaks at binding energies of 84.0 and 87.6 eV due to Au 4f_7/2_ and Au 4f_5/2_, respectively which confirmed the presence of Au^0^.^49^ The deconvoluted spectra of O 1s showed two peaks at 530.6 eV was attributed to the lattice oxygen of TiO_2_ or the metal–oxygen bond^50^ and 532.1 eV resulted from the existence of O–H bond.^51^ The Ni 3p region was used to analyse the oxidation state of nickel in the prepared photoelectrode similar to Amaya-Dueñas *et al.*^52^ and peak at 63.7 eV was observed and it was assigned to NiO.^53^

**Fig. 9:**
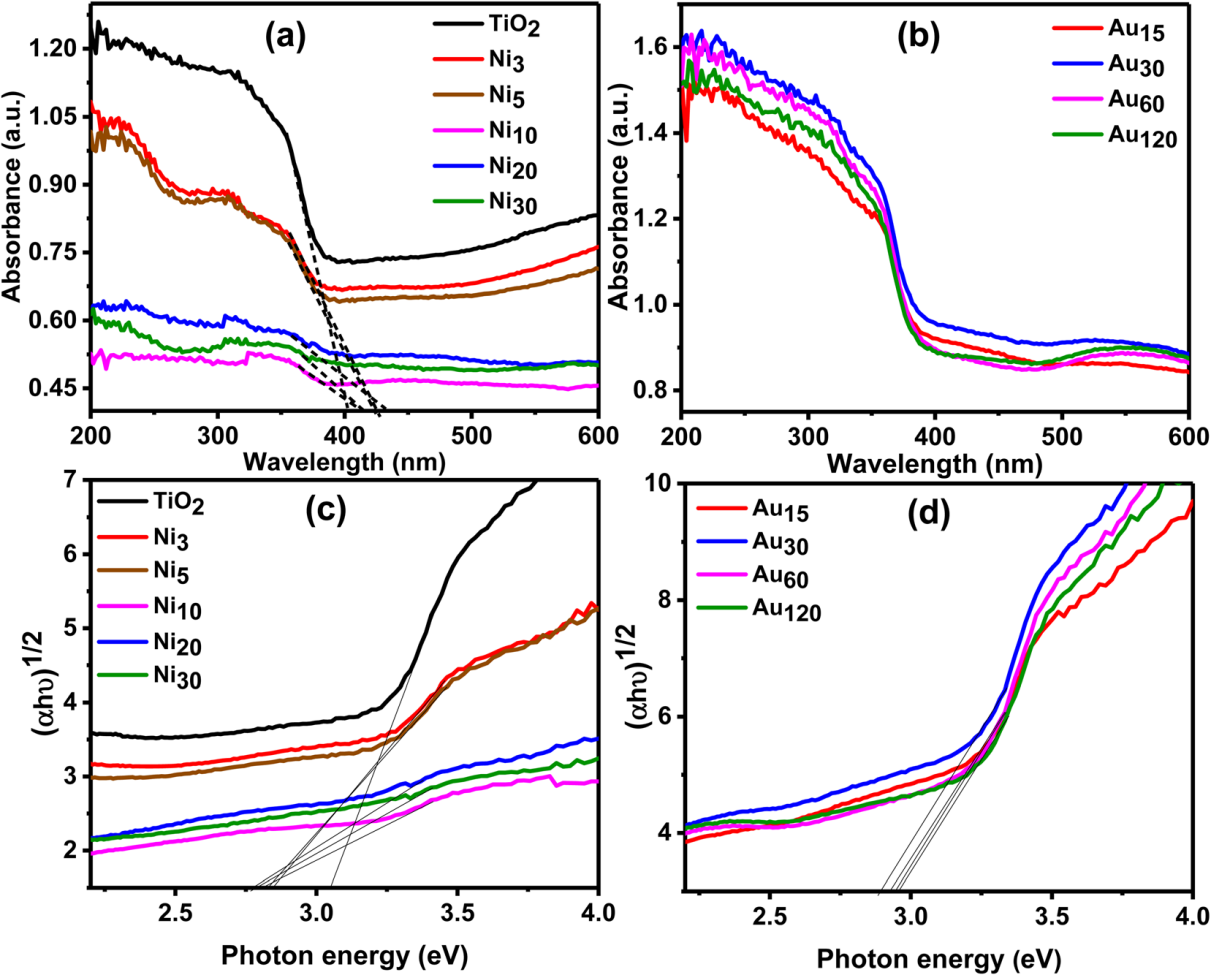
Diffuse reflectance UV-visible spectra of (a) TiO_2_NTs and Ni_*x*_/TiO_2_NTs photoanodes, and (b) Au_*y*_/Ni_20_/TiO_2_NTs photoanodes. Tauc plot of (c) Ni_*x*_/TiO_2_NTs and (d) Au_*y*_/Ni_20_/TiO_2_NTs.

Fig. 9 depict the UV-vis diffuse reflectance absorption spectra (DRS) of the pure TiO_2_NTs, Ni_*x*_/TiO_2_ and Au_*y*_/Ni_*x*_/TiO_2_NTs photoelectrodes in the range of 200–600 nm. The strong absorption observed in a wavelength region below 403 nm for the pure TiO_2_NTs is due to the intrinsic inter-band transition absorption of TiO_2_NTs.^54,55^ The light absorption edge was red shifted to the visible light for Ni_*x*_/TiO_2_NTs (Fig. 9a) photoelectrodes which can be explained by formation of a p–n heterojunction was formed across TiO_2_/NiO interface leading to electron excitation by visible light photons. Au_*y*_/Ni_20_/TiO_2_NTs (Fig. 9b) photoelectrodes showed enhanced absorption in the visible light region than pristine TiO_2_NTs while absorption peak in the range of 500–600 nm appeared in the spectrum. This peak resulted from the LSPR effect induced by gold nanoparticle.^34,56,57^ It has been reported that the LSPR effect leads to enhanced OER performance of photoanodes.^34,58^ To estimate the band gap energy of the prepared photoelectrodes, Kubelka–Munk transformation on the obtained UV-vis absorption data was used to produce Tauc plot which was represented by Fig. 9c and d. A Tauc plot was constructed by plotting (*αhν*)^1/2^*versus hν* and the band gap energy (*E*_g_) values were obtained by extrapolating linear region of the curves to meet *x*-axis^59^ and cited in Table 1. The *E*_g_ value of TiO_2_NTs was found to be 3.05 eV in accordance with the reported data of titania nanotubes.^60^ TiO_2_NTs band gap is due to Ti 3d and O 2p hybridized states where the conduction band's lowest edge being formed by Ti d_*xy*_ states and the O 2p_*x*_p_*y*_ states forms the highest edge of valence band.^14^ Ni_*x*_/TiO_2_NTs and Au_*y*_/Ni_20_/TiO_2_NTs showed a narrowed band gap suggesting photoinduction of e^−^/h^+^ pair by visible light thus maximizing utilization of solar light. The obtained results are in accordance with Oros-Ruiz *et al.* who reported reduction of band gap value of TiO_2_–P25 from 3.1 to 2.9 eV after loading of Au–NiO.^35^

**Fig. 10:**
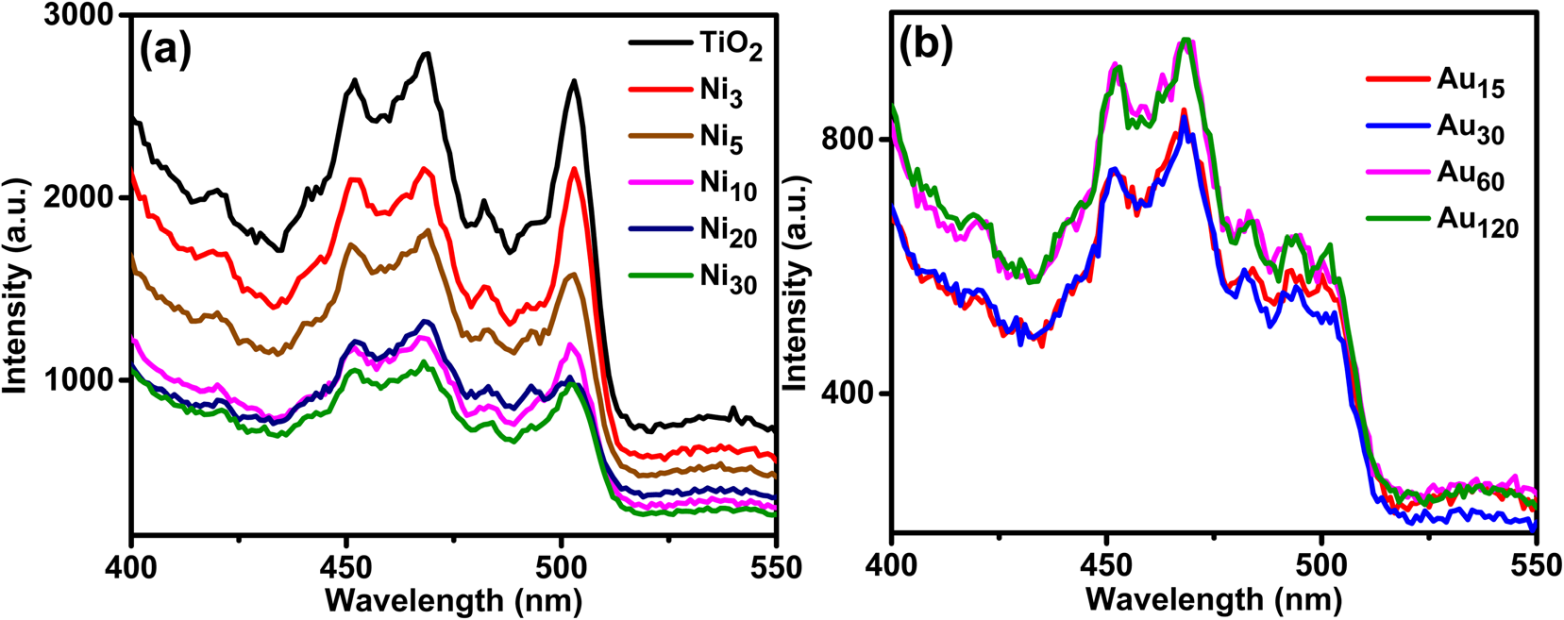
PL spectra of (a) TiO_2_NTs and Ni_*x*_/TiO_2_NTs photoanodes, and (b) Au_*y*_/Ni_20_/TiO_2_NTs photoanodes.

Photoluminescence (PL) spectra of the prepared photoelectrodes were investigated to study surface processes including photoexcited e^−^/h^+^ recombination rate and the efficiency of charge trapping and given in Fig. 10. The PL spectra of TiO_2_NTs showed the highest intensity and exhibited peaks at 420 and 452 nm assigned to self-trapped excitons^61^ where peaks at 469, 482 and 502 nm can be ascribed to oxygen vacancies and surface defects.^62^ The PL spectra of Ni_*x*_/TiO_2_NTs photoelectrodes showed the same peaks but with reduced intensity indicating lower rate of photoexcited e^−^/h^+^ recombination and improved efficiency of charge separation which is beneficial in the PEC process.^43^ Further reduction in the intensity was observed in Au_*y*_/Ni_20_/TiO_2_NTs photoelectrodes due to emerging trapping sites resulted from Au doping. The intensities of PL peaks of Ni_*x*_/TiO_2_NTs and Au_*y*_/Ni_20_/TiO_2_NTs samples were generally lower than pristine titania nanotubes. The PL intensities of Au_*y*_/Ni_20_/TiO_2_NTs were decreased in the order Au_120_ ≈ Au_60_ > Au_30_ ≈ Au_15_. It is known that PL signals of semiconductor materials result from the recombination of photoinduced charge carriers thus in general, the lower the PL intensity, the lower the recombination rate of photoinduced electron–hole pairs, and hence higher photocatalytic activity.^63^ However, the PL mechanisms of semiconductor nanoparticles are very complex, and it is not possible to extrapolate the contribution of the single factors since various parameters, as for instance particle size, recombination velocity, and presence of defects, are responsible in a different way for the shape and intensity of PL spectra. In this work, different periods were used for photo deposition of Au which might affected the particle size of Au giving rise to larger size at extended deposition duration. This could affect the rate of carriers' recombination and hence the PL intensity. Same behavior was reported by Khore *et al.*^49^ where the PL intensities of Au@TiO_2_ composites were decreased by increasing Au loading to 2% then increased when the loading reached 3% Au. Also, Lin *et al.*^64^ reported the intensity of the PL emission signals decreased with the decreasing of the Au NPs sizes indicating the radiation recombination of the photo-induced carriers were suppressed when the Au NPs became smaller.

### 3.2 Photoelectrochemical performance of the prepared photoelectrodes

**Fig. 11:**
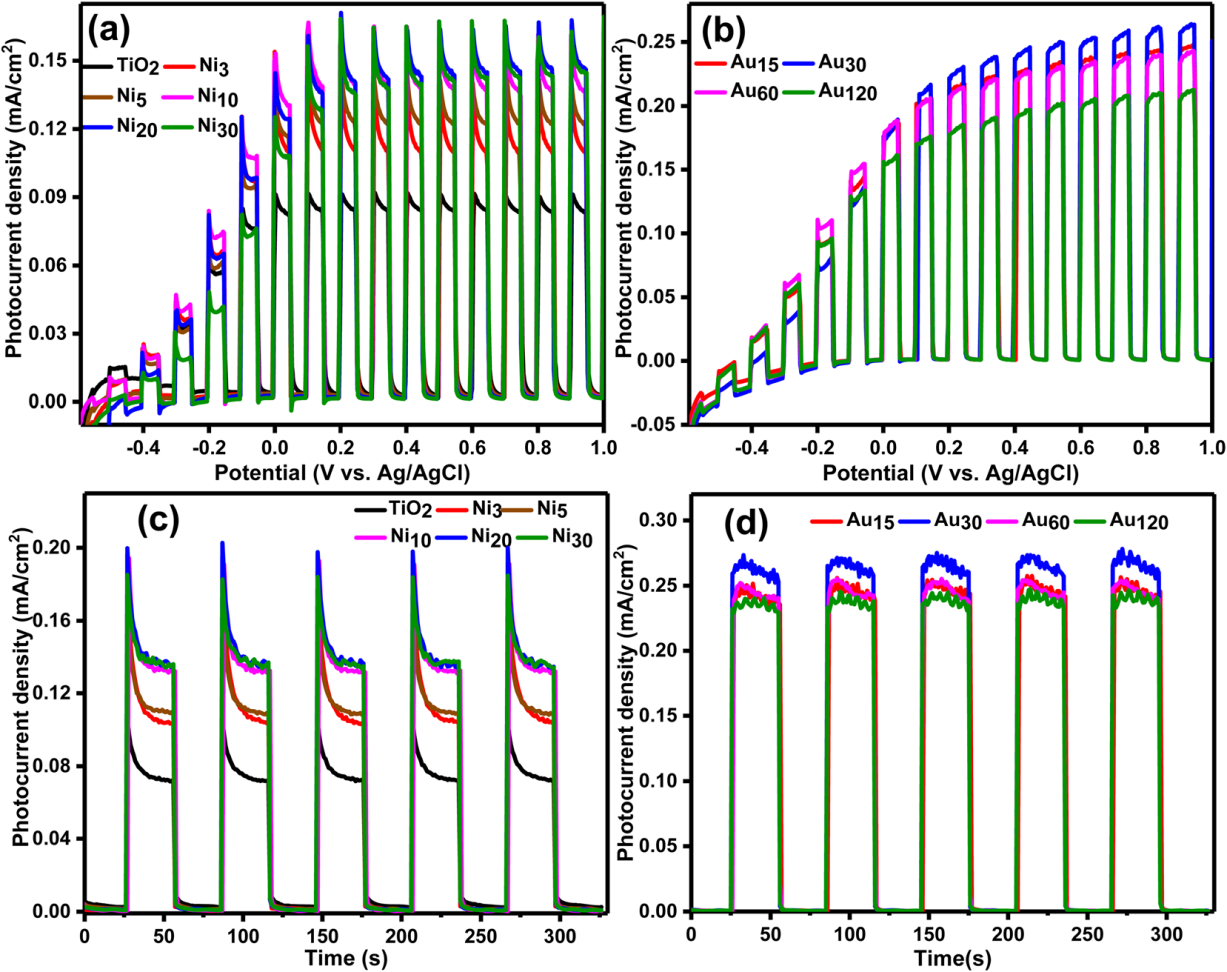
(LSV) curves at a scan rate of 10 mV for (a) TiO_2_NTs and Ni_*x*_/TiO_2_NTs photoanodes, and (b) Au_*y*_/Ni_20_/TiO_2_NTs photoanodes. The chopped light chronoamperometric measurements at a scan rate of 10 mV for (c) TiO_2_NTs and Ni_*x*_/TiO_2_NTs photoanodes, and (d) Au_*y*_/Ni_20_/TiO_2_NTs photoanodes.

Linear sweep voltammetry measurement (Fig. 11) illustrated that TiO_2_NTs, Ni_*x*_/TiO_2_NTs and Au_*y*_/Ni_20_/TiO_2_NTs photoelectrodes are all n-type semiconductors and exhibited an OER activity since under light irradiation, the photocurrent increases when the applied anodic potential is increased.^65^ TiO_2_NTs showed the lowest photocurrent density of 0.08 mA cm^−2^ attributed to the wide band gap (3.05 eV) and higher rate of recombination of photogenerated charge carriers.^27^ Ni_*x*_/TiO_2_NTs photoanodes (Fig. 11a) exhibited higher water photo-oxidation activities and higher photocurrent densities than TiO_2_NTs. This behavior is attributed to efficient separation of photogenerated charge carriers due to the Ni 3d states serving as trapping sites for photogenerated electrons from the VB which is in accordance with the PL results. The same enhancement in behavior was also reported for Ni doped titania photoanodes in PEC water splitting by many groups.^26–28,30,66^ The increase in the photocurrent density was dependent on the number of electrodeposition cycles during preparation and was found in the order Ni_3_/TiO_2_NTs < Ni_5_/TiO_2_NTs < Ni_10_/TiO_2_NTs < Ni_20_/TiO_2_NTs ≈ Ni_30_/TiO_2_NTs. The photocurrent density of Ni_20_/TiO_2_NTs was 0.14 mA cm^−2^ that is 1.75-fold of pristine TiO_2_NTs, and it can be concluded that 20 cycles of electrodeposition was efficient in producing significant enhancement in the photoanode performance. Photodeposition of gold nanoparticles boosted the OER activity of Ni_20_/TiO_2_NTs photoanode as can be seen in Fig. 11b which revealed the photocurrent density of Au_*y*_/Ni_20_/TiO_2_NTs photoanodes was higher than both pristine TiO_2_NTs and Ni_20_/TiO_2_NTs due to synergistic effect of co-doping of Ni and Au. The photocurrent densities were found in the order Au_120_/Ni_20_/TiO_2_NTs < Au_60_/Ni_20_/TiO_2_NTs < Au_15_/Ni_20_/TiO_2_NTs < Au_30_/Ni_20_/TiO_2_NTs. The photocurrent density of Au_30_/Ni_20_/TiO_2_NTs was 0.26 mA cm^−2^ that is 3.25-fold of pristine TiO_2_NTs, this enhancement of performance can be explained in light of (PL) results where Au_30_/Ni_20_/TiO_2_NTs was the most efficient photoanode in charge separation thus suppressing recombination of photogenerated charge carrier which led to enhanced photoelectrochemical performance.

Chronoamperometric measurements measured at 1 V *vs.* Ag/AgCl on the prepared photoanodes under chopped light irradiation was presented in Fig. 11. All the prepared photoanodes showed excellent solar light response as illustrated by the fast photo-response in the ON/OFF cycles and the good reproducibility. The measured photocurrent density of TiO_2_NTs was 0.07 mA cm^−2^ which is comparable with published data on TiO_2_ photoanodes.^67^ Photoanodic current spikes can be observed for TiO_2_NTs and Ni_*x*_/TiO_2_NTs photoanodes (Fig. 11c) at the onset of the potential attributed to the undesired fast recombination of photo-generated carriers at photoanode surface which limit their transport to the electrolyte. In this situation charge recombination is kinetically faster than charge transport (electrons to bulk regions or holes to the electrolyte).^65^ The measured photocurrent density of the prepared Ni_*x*_/TiO_2_NTs photoanodes was comparable with the data from LSV showing the same dependence on the number of electrodeposition cycles. Upon photoelectrodepostion of gold on Ni_20_/TiO_2_NTs, the photoanodic current spikes disappeared confirming efficient charge separation. The highest photocurrent density of Au_*y*_/Ni_20_/TiO_2_NTs photoanodes was exhibited by Au_30_/Ni_20_/TiO_2_NTs (0.26 mA cm^−2^) consistent with LSV measurements. The measured values of photocurrent density were dependant on the number of gold photo-electrodeposition cycle reaching a maximum at 30 cycles. Increase the number of cycles above that limit would cause a slight reduction in the photocurrent density probably due to creation of additional recombination sites.^67^ The observed enhanced OER activity of Au_30_/Ni_20_/TiO_2_NTs could be attributed to LSPR effect of nanometric gold which increased solar light harvesting and the synergism between Ni and Au which led to better charge separation and transportation. The obtained data of chronoamperometry was in accordance with the data obtained from optical measurements and linear sweep voltammetry.

**Fig. 12:**
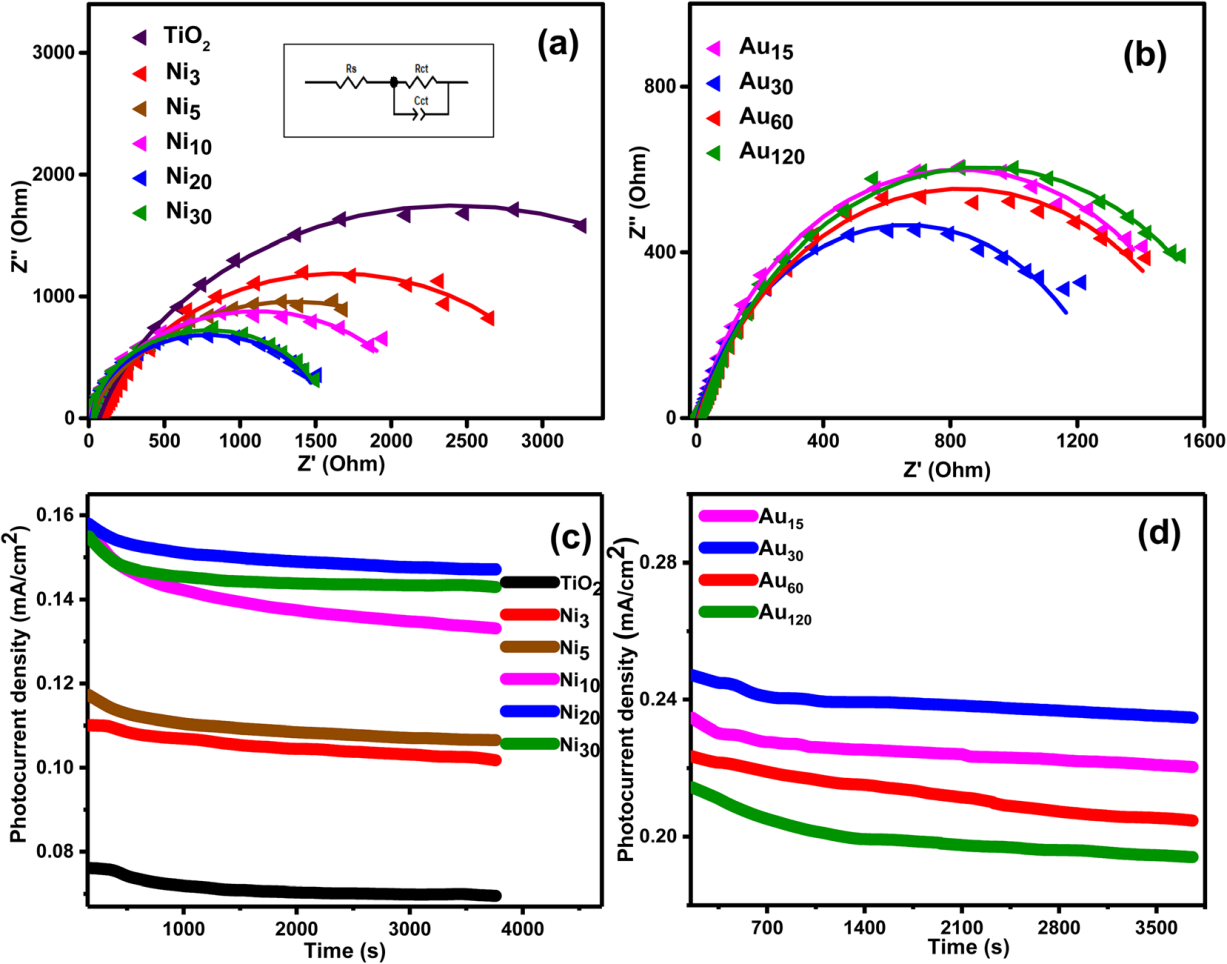
Electrochemical impedance spectroscopy plot of (a) TiO_2_NTs and Ni_*x*_/TiO_2_NTs photoanodes, and (b) Au_*y*_/Ni_20_/TiO_2_NTs photoanodes. The proposed equivalent circuit (inset (a)). The stability measurement at 1.0 V for 1 h of (c) TiO_2_NTs and Ni_*x*_/TiO_2_NTs photoanodes, and (d) Au_*y*_/Ni_20_/TiO_2_NTs photoanodes.

**Fig. 13:**
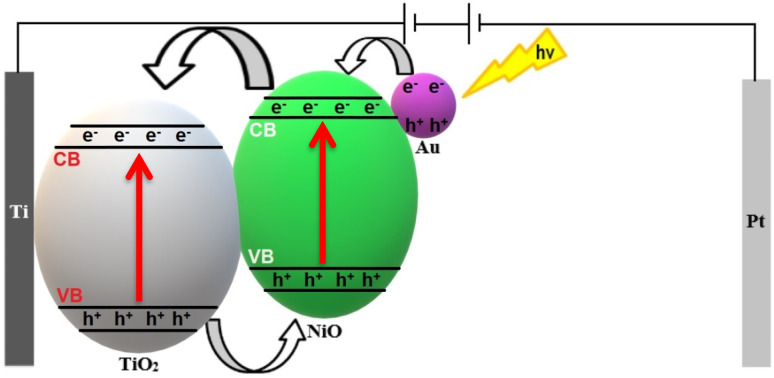
The proposed mechanism for charge transfer in Au_*y*_/Ni_20_/TiO_2_NTs photoanodes during PEC.

Electrochemical impedance spectroscopy was conducted to elucidate the charge transfer characteristics at the photoelectrode/electrolyte interface and the Nyquist plots are given in Fig. 12. It is well known that the radius of semicircle denotes the electron transfer at interface,^68^ where a larger radius generally indicates larger electron transfer resistance and lower charge separation efficiency.^58,69,70^ As shown in Fig. 12a, the semicircle radius of all Ni_*x*_/TiO_2_NTs photoanodes was smaller than that of TiO_2_NTs indicating lower charge transfer resistance, long charges lifetime and higher charge separation efficiency.^71^ Further reduction in the semicircle radius was observed in Au_*y*_/Ni_20_/TiO_2_NTs photoanodes (Fig. 12b) and Au_30_/Ni_20_/TiO_2_NTs showed the smallest charge transport resistance. The equivalent circuit diagram was suggested (inset Fig. 13a) and the values of the charge transfer resistance *R*_ct_ were calculated. The *R*_ct_ of pristine TiO_2_NTs was 4.80 kΩ which was reduced in Ni_*x*_/TiO_2_NTs photoanodes following the order Ni_20_/TiO_2_NTs (1.54 kΩ) < Ni_30_/TiO_2_NTs (1.58 kΩ) < Ni_10_/TiO_2_NTs (2.19 kΩ) < Ni_5_/TiO_2_NTs (2.54 kΩ) < Ni_3_/TiO_2_NTs (3.14 kΩ). The values of *R*_ct_ of Au_*y*_/Ni_20_/TiO_2_NTs photoanodes were Au_30_/Ni_20_/TiO_2_NTs (1.34 kΩ) < Au_15_/Ni_20_/TiO_2_NTs (1.65 kΩ) < Au_60_/Ni_20_/TiO_2_NTs (1.67 kΩ) < Au_120_/Ni_20_/TiO_2_NTs (1.75 kΩ). The reduction in *R*_ct_ values means more holes are participating in water oxidation reaction and better separation efficiency. The photoanode Au_30_/Ni_20_/TiO_2_NTs exhibited the smallest charge transfer resistance and higher charge separation efficiency in accordance with other electrochemical and optical measurement suggesting its application as efficient photoanode in PEC water splitting for H_2_ production.

The stability of the different photoanodes was determined by chronoamperometry at 1 V *vs.* Ag/AgCl under constant light irradiation and the results obtained during the first hour are shown in Fig. 12. The photocurrent density was marginally reduced for all photoanodes suggesting good stability. Comparison with other reported photoanodes (Table 2) suggested reasonably high photocurrent density of the prepared photoanode which confirmed its potential application as efficient and stable photoanode in PEC water splitting for H_2_ production.

**Table 2:** Photocurrent density comparison with other reported photoanodes

Photoanode	Electrolyte	Voltage	Photocurrent density (mA cm^−2^)	Ref.
Ag–N, S–C/TiO_2_@CC	0.5 M phosphate buffer solution	1.23 V *vs.* RHE	0.089	75
m-RGO/TiO_2−*x*_	0.1 M Na_2_SO_4_	0.2 V *vs.* SCE	0.018	76
CdSe/TiO_2_ NTs	0.2 M Na_2_S	0.5 V *vs.* SCE	0.167	77
AgVO_3_/V_2_O_5_–TiO_2_	0.2 M Na_2_SO_3_ and 0.1 M Na_2_S		0.115	78
GQDs/TiO_2_-20	0.5 M Na_2_SO_4_	0.5 V *vs.* SCE	0.072	79
Cr–S-codoped TiO_2_	1.0 M KOH	0.40 V *vs.* Ag/AgCl	0.202	80
TNT-15Ti_3_C_2_T_*x*_	0.5 M Na_2_SO_4_	0.6 V *vs.* Ag/AgCl	0.177	81
5 nm Au NPs@TNAs	1 M KOH	1.23 V *vs.* RHE	0.140	37
Au(556)/TiO_2_NTPC	—	1.23 V *vs.* RHE	0.150	82
W-h-TiO_2_	0.1 M Na_2_SO_4_	0.6 V *vs.* Ag/AgCl	0.080	16
Ni ALD/black TiO_2_	1 M KOH	0.23 V *vs.* Ag/AgCl	0.165	26
Ni–TiO_2_ nanorods	0.1 M Na_2_SO_4_	0 V *vs.* (Ag/AgCl)	0.140	66
CFTS/Ni–TiO_2_	0.1 M Na_2_SO_4_	0 V *vs.* (Ag/AgCl)	0.190	66
Au_30_/Ni_20_/TiO_2_NTs	0.5 M Na_2_SO_4_	1 V *vs.* Ag/AgCl	0.260	Current work

The following mechanisms were proposed based on the obtained results from optical and electrochemical measurements after considering the published mechanisms in literature.^1,4,10,14,56,72–74^ First for Ni_*x*_/TiO_2_NTs photoanodes; a p–n heterojunction was formed across the TiO_2_NTs (n-type semiconductor)/NiO (p-type semiconductor) interface. Upon visible light excitation charge carriers were photogenerated in both semiconductors followed by migration of electrons from the CB of NiO to the CB of TiO_2_ then through the electric circle to the Pt wire. Meanwhile the photogenerated holes travelled through the p–n heterojunction interface from TiO_2_ VB to NiO VB thus establishing efficient charge separation. The holes in NiO VB directly engaged in H_2_O oxidation reaction to produce O_2_ gas while H_2_ is produced at the Pt cathode. Second for Au_*y*_/Ni_20_/TiO_2_NTs photoanodes (Fig. 13); it was proposed that Au nanoparticles were photo-deposited on the surface of NiO forming Schottky barrier at the Au/NiO interface. Visible light excitation activated the (LSPR) effect which led to enhanced light harvesting and hot electron generation in the plasmonic Au nanoparticles. The hot electrons overcome the Schottky barrier and migrated to the CB of NiO then through the p–n heterojunction interface to the CB of TiO_2_ where it was transferred to the cathode. Accordingly, further improvement in the charge separation efficiency and higher photocurrent densities were obtained due to the favorable synergism between the p–n heterojunction and LSPR effects.

## 4. Conclusion

A series of Ni_*x*_/TiO_2_NTs and Au_*y*_/Ni_*x*_/TiO_2_NTs photoanodes were prepared and its activity toward photoelectrochemical water-splitting for H_2_ production under simulated solar light was investigated. A correlation was found between the photoanodes performance and the number of electrodeposition cycles and duration of photoreduction of gold. The photocurrent density was increased in the order Ni_3_/TiO_2_NTs < Ni_5_/TiO_2_NTs < Ni_10_/TiO_2_NTs < Ni_20_/TiO_2_NTs ≈ Ni_30_/TiO_2_NTs. The photocurrent density of Ni_20_/TiO_2_NTs was 0.14 mA cm^−2^ that is 1.75-fold of pristine TiO_2_NTs. Photodeposition of gold further boosted the OER activity of Ni_20_/TiO_2_NTs photoanode and was found to increase in the order Au_120_/Ni_20_/TiO_2_NTs < Au_60_/Ni_20_/TiO_2_NTs < Au_15_/Ni_20_/TiO_2_NTs < Au_30_/Ni_20_/TiO_2_NTs. The photocurrent density of Au_30_/Ni_20_/TiO_2_NTs was 0.26 mA cm^−2^ that is 3.25-fold of pristine TiO_2_NTs, this enhancement was attributed to LSPR effect of nanometric gold and the synergism between Ni and Au leading to better solar light harvesting, charge separation and transportation. This research may have significant implications for the synthesis of nanomaterials with improved light harvesting for photo- and photoelectrocatalytic applications in the future.

## Conflicts of interest

There are no conflicts to declare.

## Supplementary Material

RA-013-D3RA02011H-s001
