# Supplementary material for: Optimizing the performance of Auy/Nix/TiO2NTs photoanodes for photoelectrochemical water splitting
Source: RSC Adv. 2023 May 9;13(20):14018–32. doi: 10.1039/d3ra02011h (PMC10167675; doi:10.1039/d3ra02011h)
Supplement: RA-013-D3RA02011H-s001 [file RA-013-D3RA02011H-s001.pdf]

Electronic Supplementary Material (ESI) for RSC Advances.
This journal is © The Royal Society of Chemistry 2023

Supporting Information

Optimizing the Performance of $Au_y/Ni_x/TiO_2NTs$ Photoanodes for Photoelectrochemical Water Splitting

Shaimaa K. Mohamed[1,*], Amany M. A. Bashat[1], Hassan M.A. Hassan[1,*], Nahla Ismail [2], Waleed M. A. El Rouby [3,*]

[1] Department of Chemistry, Faculty of Science, Suez University,43518, Suez, Egypt

[2]Physical Chemistry Department, Centre of Excellence for Advanced Sciences, Renewable Energy Group, National Research Centre, 12311, Dokki, Giza, Egypt.

[3] Materials Science and Nanotechnology Department, Faculty of Postgraduate Studies for Advanced Science (PSAS), Beni-Suef University, 62511 Beni-Suef, Egypt

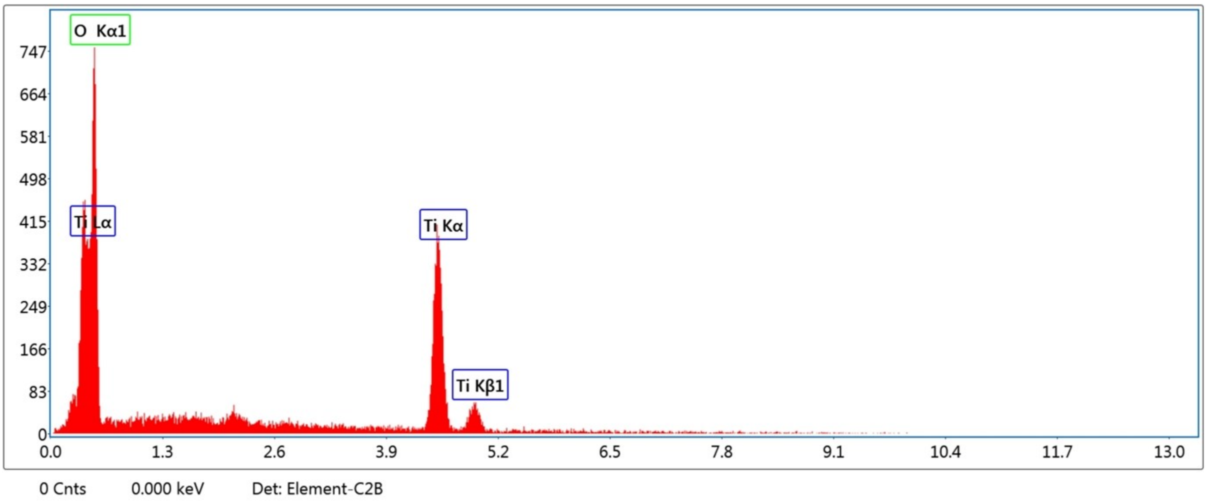

Fig. S1: The EDS elemental analysis of TiO_2NTs.

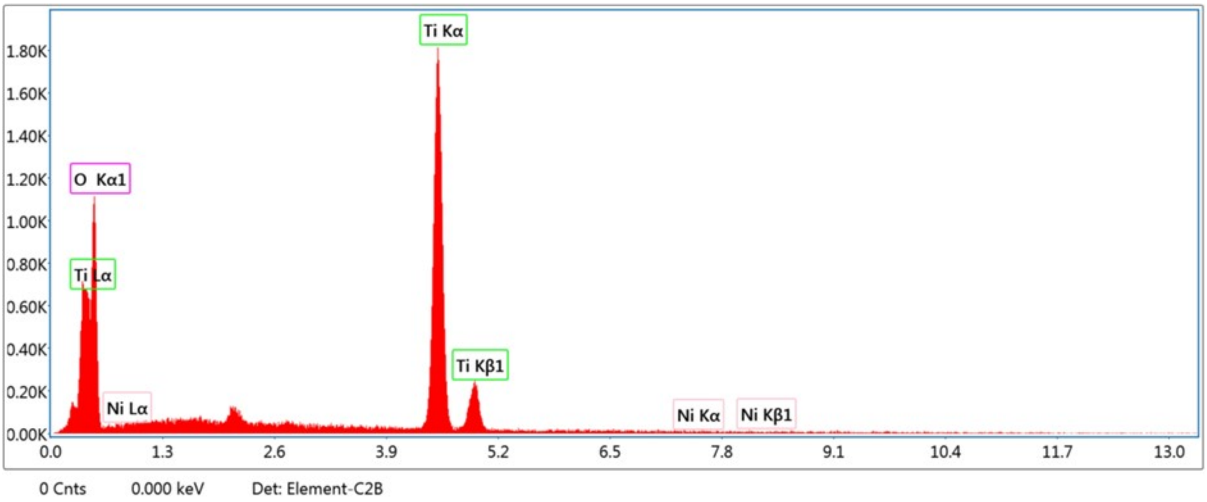

Fig. S2: The EDS elemental analysis of Ni_3/TiO_2NTs.

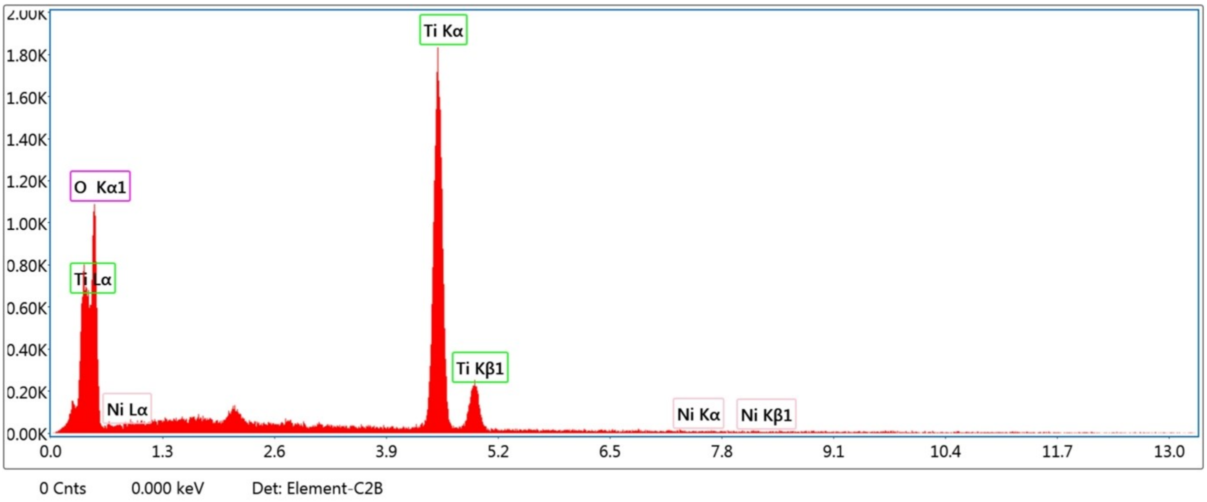

Fig. S3: The EDS elemental analysis of Ni_5/TiO_2NTs.

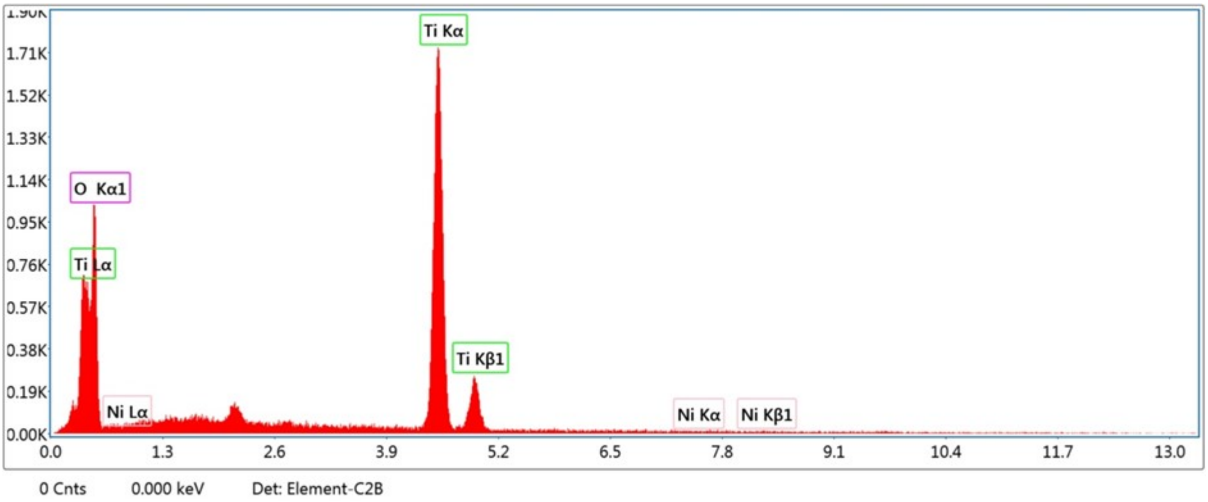

Fig. S4: The EDS elemental analysis of Ni_{10}/TiO_2NTs.

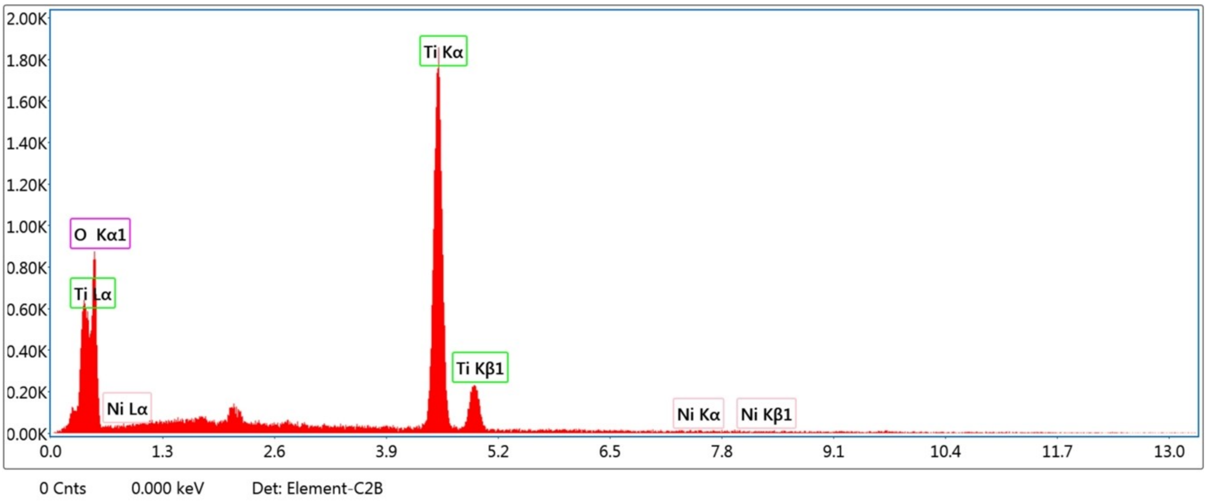

Fig. S5: The EDS elemental analysis of Ni_{20}/TiO_2NTs.

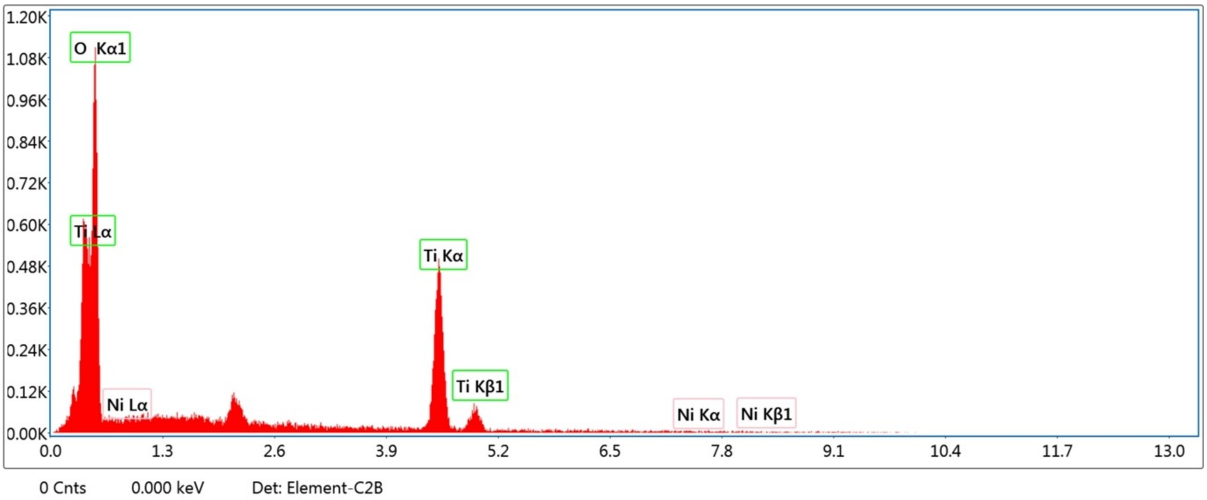

Fig. S6: The EDS elemental analysis of Ni_{30}/TiO_2NTs.

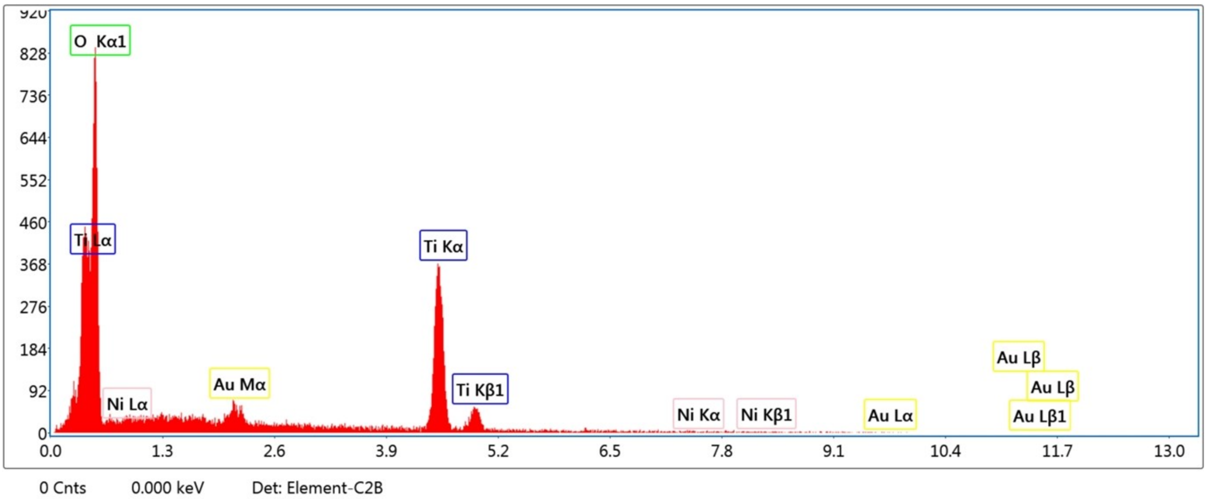

Fig. S7: The EDS elemental analysis of $Au_{15}/Ni_{20}/TiO_2NTs$.

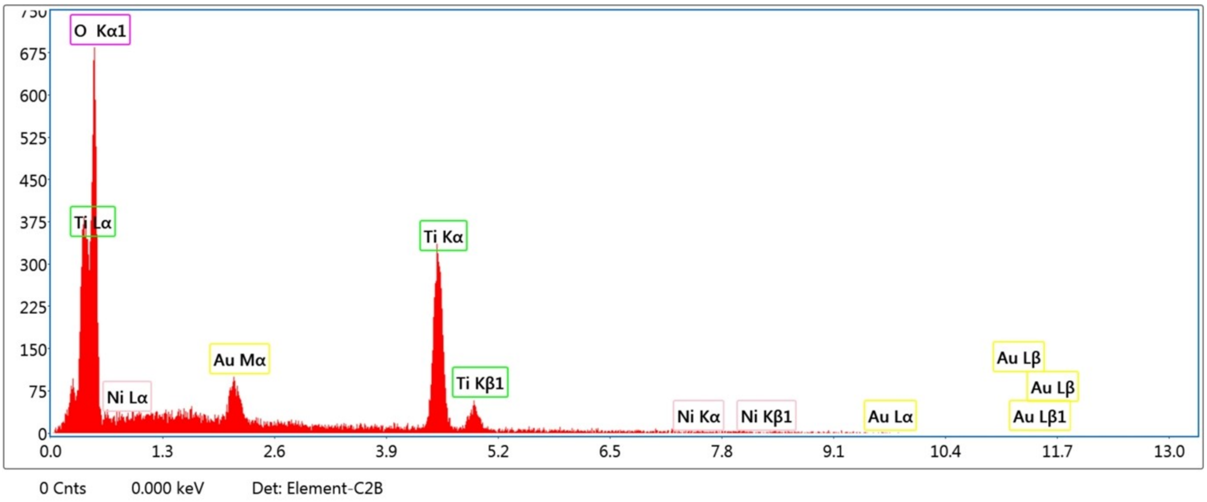

Fig. S8: The EDS elemental analysis of $Au_{30}/Ni_{20}/TiO_2NTs$.

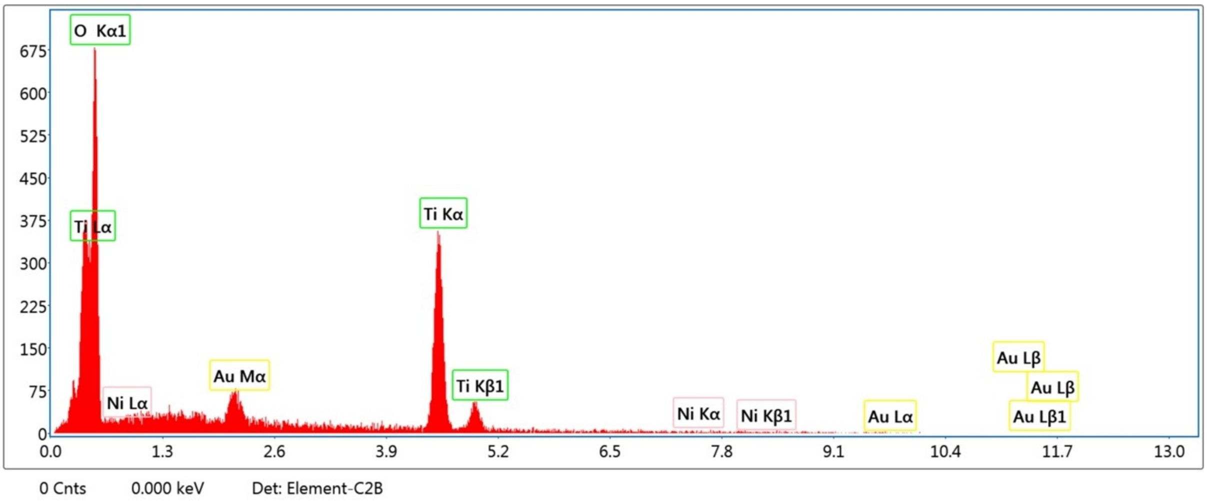

Fig. S9: The EDS elemental analysis of $Au_{60}/Ni_{20}/TiO_2NTs$.

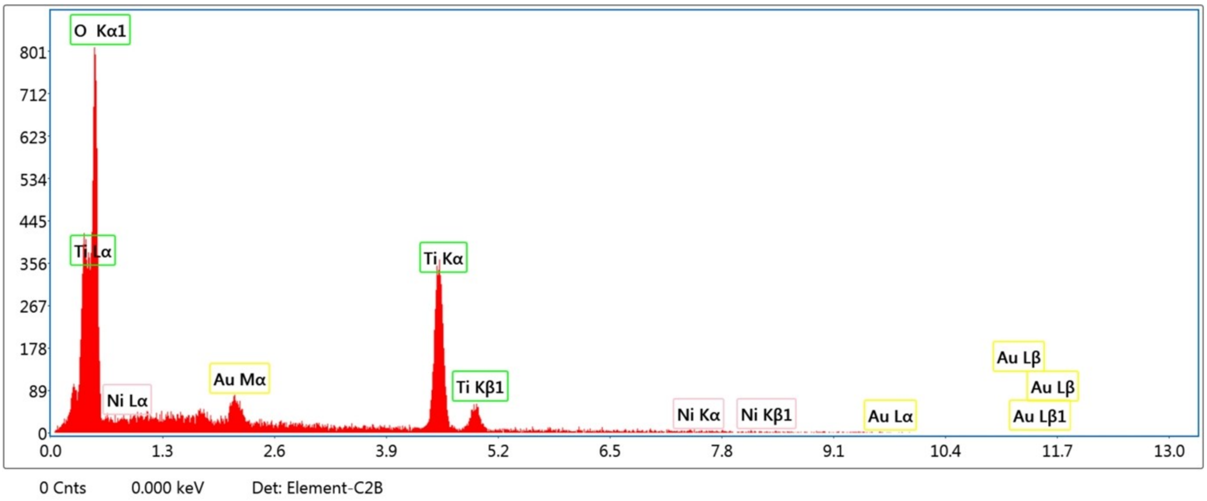

Fig. S10: The EDS elemental analysis of $Au_{30}/Ni_{20}/TiO_2NTs$.
